# Nasal Airway Respiration Anti-tic (NARA) method in pediatric Tourette syndrome: a retrospective case series

**DOI:** 10.1186/s12887-026-06856-w

**Published:** 2026-04-18

**Authors:** Takanobu Kaido, Hidehiro Hirabayashi, Nagako Murase, Kaori Kaido, Yasuko Sawai, Takafumi Sakakibara, Homare Akahane, Tatsuo Shimokawara, Kiyoshi Nagata

**Affiliations:** 1 Department of Neurosurgery, National Hospital Organization Nara Medical Center, 2-789 Shichijo, Nara City, Nara 630-8053 Japan; 2 Department of Health and Nutrition, Anatomy and Physiology Laboratory, Osaka Shoin Women’s University, Higashiosaka, Japan; 3 Department of Rehabilitation, Naramachi Rehabilitation Hospital, Seiwakai Medical Corporation Group, Nara, Japan; 4 Department of Neurology, National Hospital Organization Nara Medical Center, Nara, Japan; 5 Higashihanazono Life Support Center, Higashiosaka, Japan; 6 Department of Child Neurology, National Hospital Organization Nara Medical Center, Nara, Japan; 7 Department of Otolaryngology, National Hospital Organization Nara Medical Center, Nara, Japan

**Keywords:** Tourette syndrome, Tics, Breathing, Lip closure, Immediate relief, Long-term improvement

## Abstract

**Background:**

Tourette syndrome (TS) often impairs social and academic functioning in children due to disruptive motor and vocal tics. Minimally invasive and patient-driven interventions are desirable, yet existing treatments do not always provide rapid or sustained relief. This study evaluates the clinical outcomes of the Nasal Airway Respiration Anti-tic (NARA) method—a non-invasive, lip-closed nasal breathing technique—for children with TS.

**Methods:**

In this uncontrolled retrospective case series, we identified all 8 consecutive pediatric patients (5 boys, 3 girls; ages 6–10) meeting DSM-5 criteria for TS who presented with habitual mouth breathing between 2019 and 2021. All patients were free of tic-suppressing medications at baseline. Patients had a chronic disease course (mean duration of illness: 2.5 years). No eligible patients were excluded. Patients were instructed to practice the NARA method (3–5 s nasal inhalation, 2s breath-holding, and 6–10 s exhalation with firm lip closure) for 2 min, 3 times daily. Tic severity was assessed using the Yale Global Tic Severity Scale (YGTSS) and a modified Rush Video Rating Scale (mRVRS) at baseline and follow-up (mean 18.4 months).

**Results:**

At the initial visit, a 120-second trial of the NARA method significantly reduced tic frequency (*p* = 0.038) and severity (*p* = 0.025). Long-term follow-up demonstrated notable improvements in YGTSS motor and vocal subscales (14.4 ± 1.7 to 2.4 ± 1.2; 13.6 ± 2.3 to 3.6 ± 1.3, respectively). Three children who had refused school attendance due to severe tics returned to regular classes. Crucially, video analysis confirmed a “carry-over effect,” showing sustained tic reduction even during natural, resting respiration (without the maneuver) at the final assessment.

**Conclusions:**

The NARA method was associated with substantial tic reduction in this small cohort. We hypothesize that the intense physiological sensory input from nasal respiration may function as a “primary reward” that activates the thalamic centromedian nucleus to overshadow the urge-to-tic cycle. Clinically, the method’s success relied on active parental support. While placebo effects cannot be entirely ruled out in this uncontrolled study, the immediate “On/Off” reversibility of tics upon lip release implies a specific physiological antagonism distinct from simple distraction. Future prospective controlled trials are warranted to validate these preliminary findings.

**Supplementary Information:**

The online version contains supplementary material available at 10.1186/s12887-026-06856-w.

## Introduction

Tourette syndrome (TS) is a chronic neurodevelopmental disorder involving both motor and vocal tics, with onset typically in childhood [1]. It affects approximately 1% of the population [2]. Children with TS often experience social stigma, bullying, and academic disruptions. Clinically, tics are often preceded by uncomfortable “premonitory urges,” and the execution of tics provides temporary relief from these sensations. This cycle suggests that tics may function as a “negative reinforcement” or a maladaptive reward-seeking behavior to alleviate internal tension [3, 4]. Pathophysiologically, TS is linked to dysfunction in the cortico-striato-thalamo-cortical (CSTC) circuits, involving abnormalities in inhibitory control and dopaminergic reward processing [5, 6]. Although antipsychotics and behavioral therapies like Comprehensive Behavioral Intervention for Tics (CBIT) can alleviate symptoms [2], they sometimes exhibit delayed onset or insufficient efficacy. Deep brain stimulation (DBS) targeting the thalamic centromedian (CM) nucleus—a key node in the reward and attention system—has shown promise in adults [7, 8], yet remains a highly invasive option that is seldom considered for children.

Recent studies indicate that habitual mouth breathing associated with incompetent lip sealing is prevalent among children [9]. Physiologically, nasal respiration is known to entrain limbic brain oscillations and modulate cognitive and emotional networks, whereas mouth breathing bypasses these potential regulatory signals [10]. This raises the possibility that correcting the respiratory route could influence the neural circuits underlying tics. Our prior research in adults with persistent TS found that adopting a closed-mouth nasal breathing pattern produced immediate and profound tic reduction [11]. We termed this phenomenon the Nasal Airway Respiration Anti-tic (NARA) effect. However, whether younger patients with TS would enjoy similar or even more pronounced benefits remained unclear.

This retrospective case series examines 8 elementary school-aged children with TS who were instructed in the NARA method and followed over a mean of 18.4 months. We evaluated both immediate changes in tic frequency—observed under videotaped conditions at the initial visit—and longer-term improvements, focusing on the potential for re-engagement in normal school activities. Our aim was to determine if closed-mouth nasal deep breathing could yield practical, clinically significant outcomes for children with TS, potentially by modulating the underlying neurophysiological drive to tic.

## Case series

### Patients and methods

#### Study design and participants

This uncontrolled retrospective case series included all 8 consecutive pediatric patients (5 boys, 3 girls; ages 6–10 at initial assessment) with TS who initially visited the National Hospital Organization Nara Medical Center between March 2019 and May 2021 (Table 1). No eligible patients were excluded during this period. Diagnosis was confirmed by a board-certified neurosurgeon (T.K.) and a child neurologist according to DSM-5 criteria [12]. Specifically, all patients exhibited multiple motor and 1 or more vocal tics persisting for more than 1 year (mean illness duration: 2.5 years), confirming the chronic nature of the disorder and ruling out provisional tic disorders. All presenting patients exhibited habitual mouth breathing, as documented through clinical observation and parental reports. Although exclusion criteria were defined as severe intellectual disability or coexisting psychiatric conditions (e.g., psychosis) that might preclude reliable follow-up, no patients met these criteria. We collected data at baseline and final follow-up, and the Institutional Review Board approved this study in accordance with the Declaration of Helsinki.

**Table 1:** Patient characteristics and outcomes of tic severity

					YGTSS					
					Tic scores					
		Age (years)			Motor		Phonic		Impairment	
Patient	Sex	Onset	First Visit	Follow-up months	First Visit	Last Visit	First Visit	Last Visit	First Visit	Last Visit
1	F	3	10	28	15	0	14	0	30	0
2	F	6	6	23	12	0	6	0	30	0
3	M	7	10	25	11	4	22	5	40	0
4	F	5	7	22	12	0	18	5	20	10
5	M	9	10	20	23	7	22	11	40	10
6	M	9	10	14	8	0	9	4	10	0
7	M	4	8	8	14	0	12	4	20	0
8	M	8	10	7	20	8	6	0	30	10

*YGTSS* Yale Global Tic Severity Scale

#### Procedures and concurrent treatments

Detailed medication histories were reviewed. Notably, at the time of the initial NARA instruction (baseline), all 8 patients were free of tic-suppressing medications (Table 2). Although some patients had a history of pharmacotherapy (e.g., haloperidol, aripiprazole, risperidone) at referring hospitals, these had been discontinued prior to study entry due to inefficacy or adverse events. During the follow-up period (March 2019 to May 2021), 6 patients remained medication-free, while 2 patients required pharmacological intervention later in the clinical course. Patient 5 initiated pimozide in March 2020 and switched to low-dose haloperidol (0.1–0.3 mg/day) in March 2021 due to symptom fluctuations. However, this switch occurred only 2 months prior to the final assessment (May 2021), indicating that the sustained improvement observed over the preceding 2 years was largely independent of this medication. Most notably, Patient 1 achieved complete tic remission (YGTSS total score 0) by December 2020 without medication. Although levetiracetam (1000 mg/day) was initiated in late December 2020 by a child neurologist (T.S.), this was prescribed specifically for epilepsy (EEG abnormalities) and parasomnia, not for tics, and was started only after the tics had already resolved. No other behavioral interventions (e.g., CBIT) were concurrently administered.

**Table 2:** Medication status of patients during the study period (March 2019 – May 2021)

Patient	Previous Medication History (Discontinued before Baseline)	Medication Status at Baseline (NARA Initiation)	Changes During Follow-up Period (until May 2021)
1	None	None	Achieved tic remission (YGTSS 0) in Dec 2020. Started Levetiracetam (Dec 2020) for epilepsy and parasomnia, after tic remission.
2	Herbal medicine (Yokukansan)	None	None
3	Haloperidol, Aripiprazole	None	None
4	Haloperidol, Risperidone, Aripiprazole	None	None
5	Aripiprazole, Guanfacine, Atomoxetine	None	Started Pimozide (Mar 2020). Switched to low-dose Haloperidol (0.1–0.3 mg/day) in Mar 2021 (only 2 months prior to study end).
6	None	None	None
7	None	None	None
8	None	None	None

#### Intervention: deep slow nasal respiration (NARA method)

**Fig. 1:**
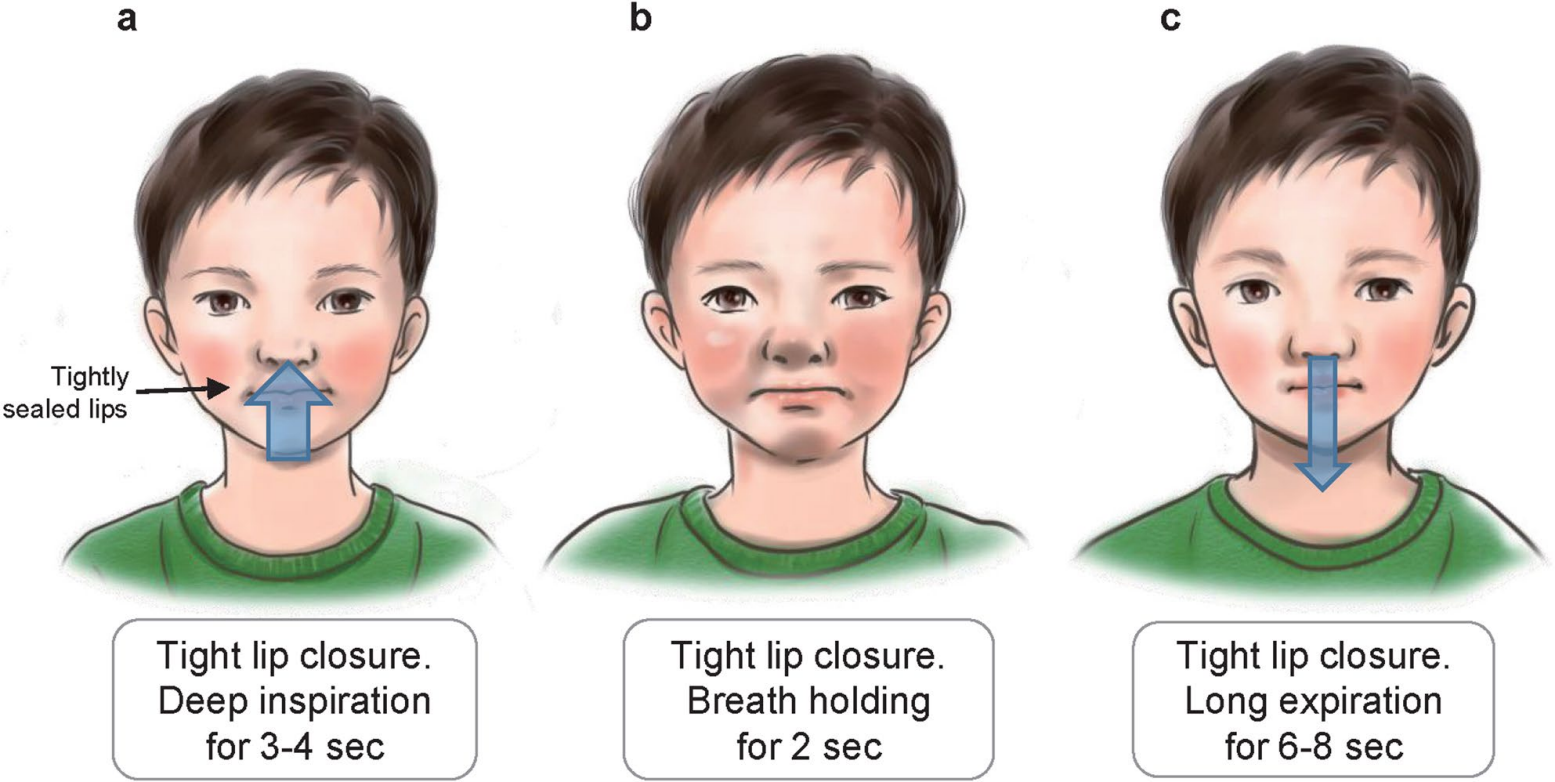
Nasal Airway Respiration Anti-tic (NARA) Method steps. **a** Inhale through the nose for 3–4 s while maintaining tight lip closure. **b** Hold the breath for 2 s. **c** Exhale through the nose for 6–8 s, continuing to keep the lips sealed

The NARA method was adapted from our previous adult study [11] to be child-friendly. Patients were instructed to (1) close their lips tightly, (2) inhale slowly through the nose for 3–4 s, (3) hold their breath for 2 s, and (4) exhale slowly through the nose for 6–8 s (Fig. 1). They were specifically advised not to attempt tic suppression during breathing. Initial training occurred in the clinic, with a 120-second demonstration captured on video. Families were then asked to practice the same method at home for 2 min, 3 times daily (morning, afternoon, and before bedtime).

Children were instructed to practice the NARA method daily at home as a patient-centered therapy, with supportive supervision and encouragement provided by their parents or caregivers. Parental involvement was emphasized as an essential factor for consistent adherence, successful self-management, and overall treatment efficacy.

#### Primary and secondary outcome measures

- Primary Outcome: Yale Global Tic Severity Scale (YGTSS) [13]. Motor and vocal tic subscales each range from 0 to 25 points (with their sum constituting the Total Tic Score, maximum 50 points), while the impairment scale ranges from 0 to 50, giving a total possible score of 100.
- Secondary Outcome: Objective tic severity was assessed using the Modified Rush Video-Based Tic Rating Scale, utilizing the scoring protocol validated by Goetz et al. (1999) [14]. While the original protocol recommends longer recording intervals, we standardized the recording duration to 120 s per condition to ensure feasibility within a busy outpatient setting and to minimize patient fatigue, focusing on capturing the high-frequency tic phenomenology. Videos were recorded under 2 conditions at the initial visit—(1) normal (habitual) respiration and (2) NARA respiration—and once again at final follow-up in each participant’s normal state.

#### Statistical analysis

A paired t-test was used to compare immediate changes (habitual vs. NARA) in tic frequency and severity, as well as baseline vs. follow-up YGTSS changes. For comparisons across 3 time points (initial habitual, initial NARA, final habitual), repeated-measures analysis of variance on ranks was performed. Statistical significance was set at *p* < 0.05. Data are reported as mean ± standard error of the mean (SEM). Analyses were conducted using SigmaPlot 14.0 (Systat Software, Chicago, IL, USA).

## Results

### Baseline characteristics

All 8 children were in elementary school (mean age 8.9 ± 0.6 years; range 6–10) and had been diagnosed with TS at least 1 year earlier. Three patients (Patients 3, 5, and 6) refused school attendance at initial presentation, citing overwhelming tic severity and social embarrassment (Table 1). The mean age at tic onset was 6.4 ± 0.8 years, and the mean duration of illness prior to the first visit was 2.5 ± 2.0 years. All patients met the DSM-5 criteria for Tourette Syndrome (duration > 1 year).

### Initial tic severity

- YGTSS: motor 14.4 ± 1.7, vocal 13.6 ± 2.3, social impairment 27.5 ± 3.7.
- mRVRS: 7.3 ± 1.5 (mean), indicating moderate severity overall.
- Average tic count: 10.5 ± 3.2 per minute on a 120-second video.

All children displayed pronounced mouth breathing at rest and had difficulty keeping their lips sealed for more than a few seconds initially.

### Immediate impact of NARA at initial visit

When the NARA method was demonstrated, we immediately recorded a significant decrease in tic frequency (4.0 ± 1.7 per minute, *p* = 0.038) and mRVRS score (3.7 ± 1.2, *p* = 0.025). Parents reported surprise at how quickly tics subsided once the children maintained proper lip closure and focused on nasal breathing.

### Home practice and clinical course

Families were instructed to continue the NARA routine at home. Although children occasionally found it challenging to sustain lip closure beyond 30–60 s in the early weeks, progress was typically evident by 1–2 months. Children who practiced more frequently—especially those with higher initial tic severity—often reported feeling an improved sense of control over impending tics.

### Case illustration (Patient 5)

A 10-year-old boy, symptomatic since age 6, had severe tics including vocal outbursts (“Ah!” and “Da-da-da!”) and body jerks that prevented him from staying in class. Initially, he could manage only 20 s of continuous nasal breathing without opening his mouth. After 4 weeks, he succeeded in sustaining lip-closed respiration for 1 full minute. By the 3-month mark, he gradually returned to partial school attendance, applying NARA breathing whenever he felt an oncoming tic wave. At the final (20-month) follow-up, he had resumed full-time classes and showed a substantial reduction in YGTSS (motor from 22 to 7; vocal from 22 to 11; social impairment from 40 to 10).

### Final follow-up outcomes

At a mean of 18.4 months follow-up, substantial improvements persisted even under normal (habitual) breathing. Three children who had initially refused school due to severe tics were once again attending classes regularly. Overall:

- Motor tics: 14.4 ± 1.7 → 4.3 ± 1.8 (p < 0.001)
- Vocal tics: 13.6 ± 2.3 → 4.1 ± 2.2 (p < 0.001)
- Social impairment: 27.5 ± 3.7 → 6.3 ± 2.6 (p = 0.001)

**Fig. 2:**
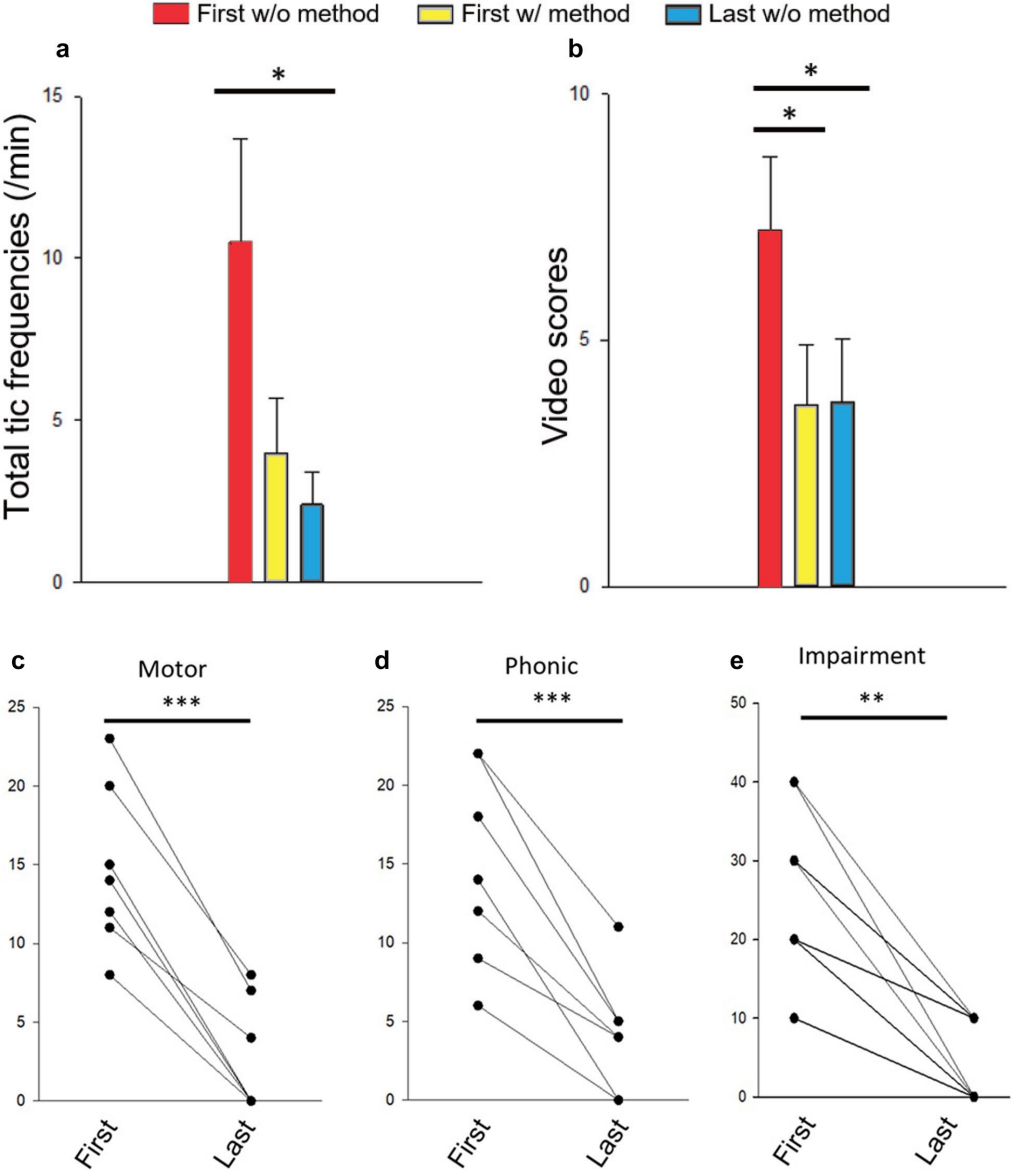
Immediate and long-term benefits of the NARA method. **a** Tic frequency and (**b**) mRVRS scores measured under 3 conditions: Initial normal breathing, initial NARA session (120 s), and final follow-up normal breathing. *P < 0.05 indicates statistical significance (repeated-measures analysis of variance on ranks). **c**-**e**. Long-term outcomes are shown by motor (**c**) and phonic (**d**) tic scores as well as social impairment (**e**) of YGTSS at the first and last visits. YGTSS, the Yale Global Tic Severity Scale. *P<0.05; **P<0.01; ***P<0.001; n.s., not significant (paired t-test)

**Fig. 3:**
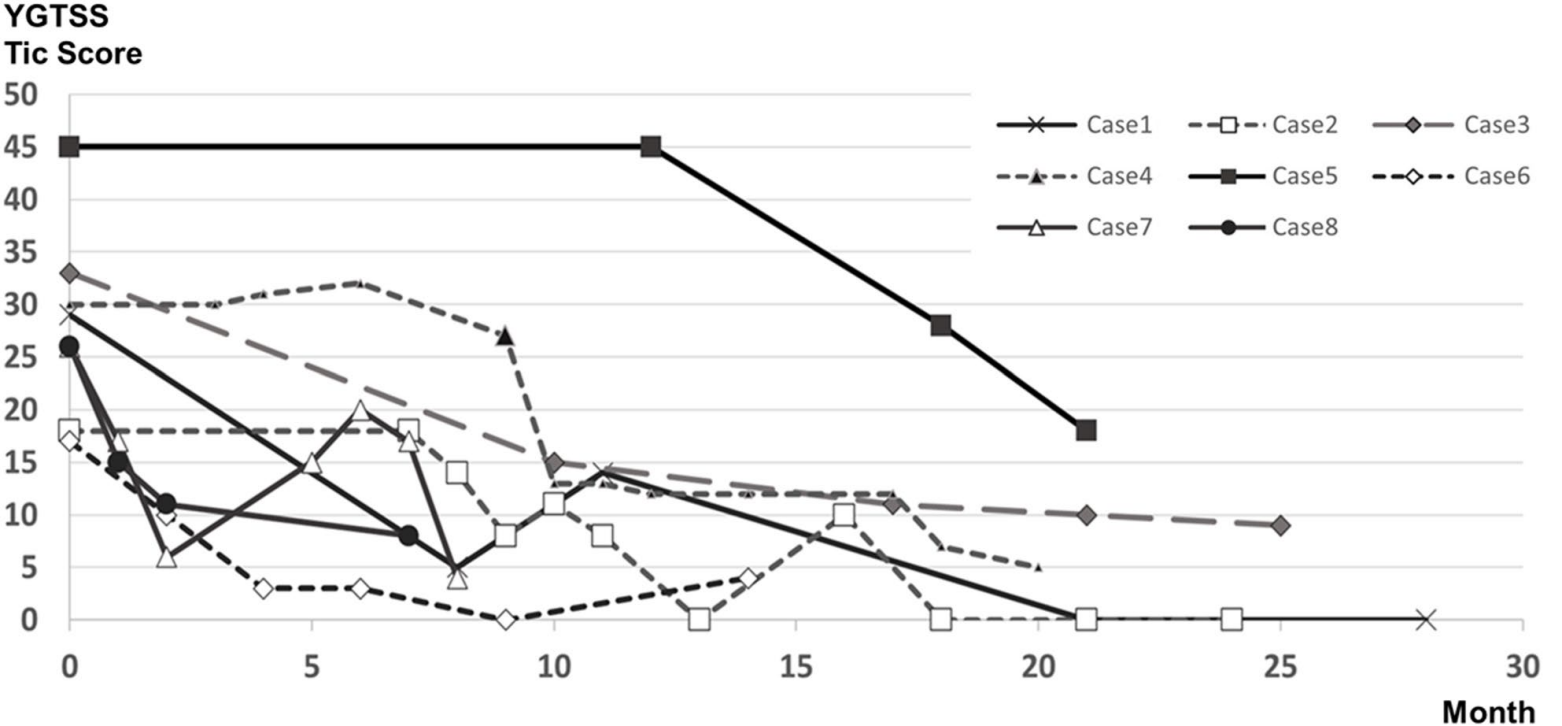
Longitudinal trajectory of tic severity for individual patients. Changes in the Yale Global Tic Severity Scale (YGTSS) Total Tic Score (sum of motor and vocal tic subscales, maximum 50 points) for each of the 8 patients over the follow-up period. The x-axis represents months since the initiation of the NARA method, with Month 0 indicating the baseline clinical visit. Note that despite individual fluctuations consistent with the natural waxing and waning of tics, all patients exhibited a progressive and sustained decline in tic severity. Importantly, at baseline (Month 0), these patients had already had a mean age at onset of 6.4 years and a mean illness duration of 2.5 years, indicating remission of a chronic condition rather than spontaneous resolution of a transient tic disorder

Likewise, the final video recordings showed an average tic frequency of 2.4 ± 1.0 per minute, and an mRVRS score of 3.8 ± 1.3 (Fig. 2). Furthermore, the longitudinal trajectory of tic severity for each patient, assessed by the YGTSS Total Tic Score, demonstrated a progressive and sustained decline over the follow-up period, despite minor individual fluctuations (Fig. 3). Even though the children were not consciously performing the NARA technique at the final video, they had evidently internalized the practice enough that their default mouth posture was less open, contributing to fewer tics in spontaneous daily life (Video 1).

## Discussion

### Brief summary

This study supports the notion that simple, closed-mouth nasal respiration can dramatically reduce tic frequency in children with Tourette syndrome, both immediately and over the long term. The NARA method harnesses deliberate lip closure, slow nasal inhalation, and regulated exhalation—a technique that not only provides rapid symptom relief but also fosters improved autonomic and sensory regulation when practiced consistently at home.

### Comparison with previous studies

Our earlier report indicated similar short-term benefits in adults (Kaido et al., 2020). The present findings expand on that by demonstrating robust effects in a younger cohort, emphasizing a strong potential for integration into pediatric care. Additionally, the prevalence of mouth breathing with incompetent lip sealing reported among children [9] underscores the potential broader applicability of interventions aimed at improving lip closure and nasal breathing.

### Physiological mechanisms: anatomical antagonism and reward overshadowing (hypothesis)

While the precise mechanisms underlying the NARA method remain to be fully elucidated, we propose 2 synergistic hypotheses involving anatomical interaction and neurophysiological reward processing. Specifically, we hypothesize that ‘Anatomical Antagonism’ provides an immediate, physical blockade of vocal tics, while ‘Reward Overshadowing’ concurrently addresses the underlying neurophysiological drive, promoting long-term habituation and structural circuit changes.

**Fig. 4:**
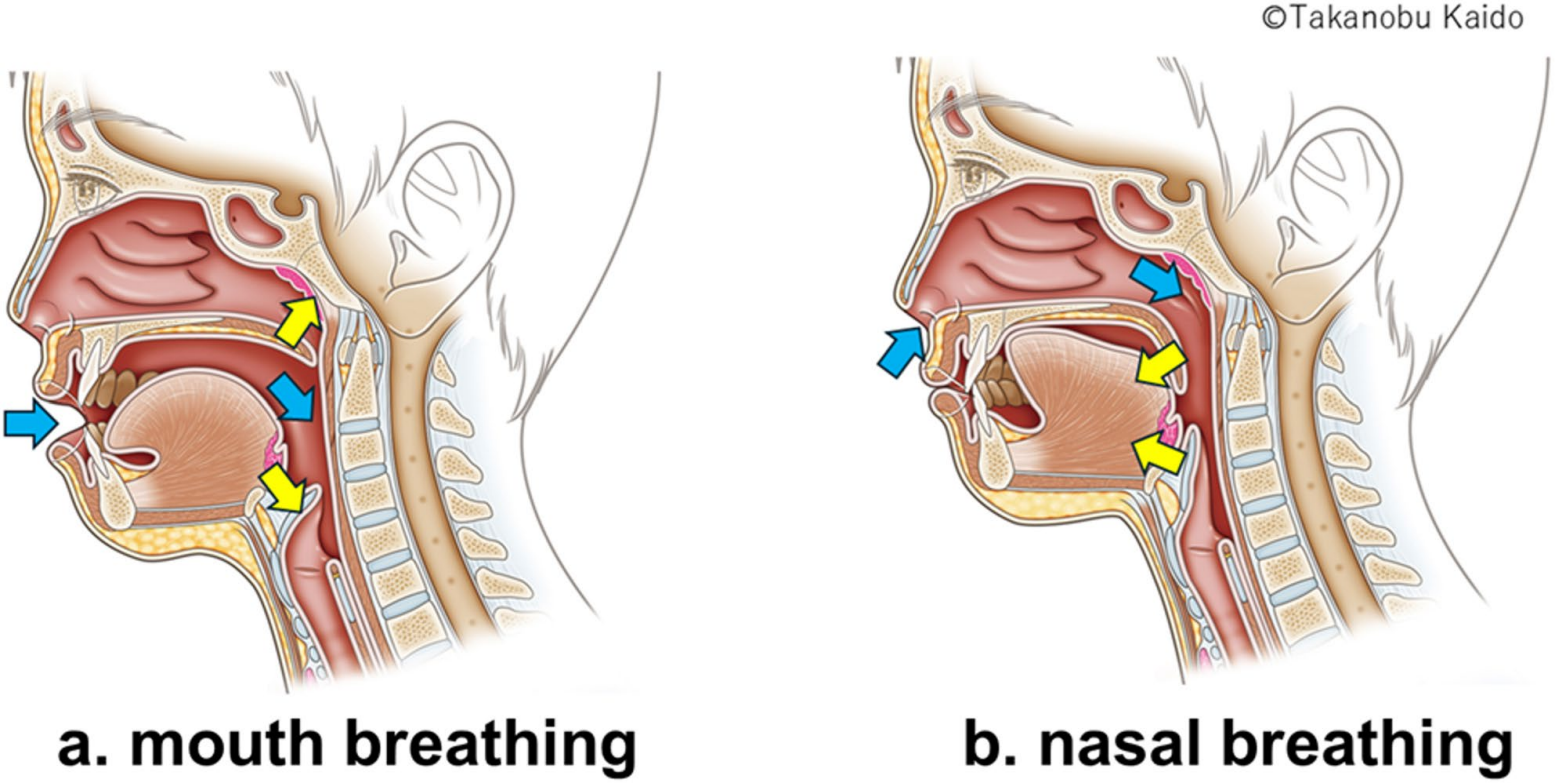
Anatomical differences between mouth breathing and nasal breathing. Blue arrows indicate airflow during inspiration. Yellow arrows illustrate movements of the soft palate and epiglottis. **a** Mouth breathing: the oral airway is maintained open, saliva accumulation increases, the epiglottis moves downward to prevent aspiration, and the soft palate elevates, blocking the nasal airway. **b** Nasal breathing: the soft palate descends to close the oral airway and open the nasal airway. The epiglottis is elevated, enhancing airway patency and promoting stable respiration

First, regarding the anatomical aspect, habitual mouth breathing creates an environment prone to tic generation. As established in anatomical literature, mouth opening triggers deglutition and utterance mechanisms [15]. Specifically, contraction of the levator veli palatini muscle posteriorly moves the soft palate to widen the oral airway, narrow the nasal airway, and lower the epiglottis (Fig. 4a) [11]. Moreover, the genioglossus and mylohyoid muscles contract to anteriorly move the tongue [16]; concurrently, the tongue apex drops to the mouth floor, increasing masticatory muscle tension [17]. We propose that this increased oral and laryngopharyngeal muscle tension induces hypersensitivity, serving as a peripheral trigger for tics [18]. In contrast, the NARA method enforces tight lip closure, which mechanically reverses this state. Visualized in our fluoroscopic study [11], this maneuver repositions the soft palate downward and elevates the epiglottis (Fig. 4b), physically separating the respiratory tract from the alimentary tract. We speculate that this anatomical reconfiguration acts as a physical “antagonist” to the specific muscle contractions involved in tics. Although we did not perform real-time imaging to confirm these internal movements in the current pediatric cohort, the immediate cessation of tics upon lip closure suggests that stabilizing the upper airway may physically or reflexively inhibit the “tic trigger.”

Second, and perhaps more critically, we propose a neurophysiological mechanism termed “Reward Overshadowing.” Clinically, tics often function as a “micro-reward” to relieve aversive premonitory urges (negative reinforcement) [3, 19]. We hypothesize that the intense sensory input provided by deep nasal breathing functions as a survival-related “Primary Reward.” Crucially, neuroimaging studies have demonstrated that the relief of air hunger is not merely the cessation of discomfort but is processed as a distinct, positively valenced rewarding sensation in the limbic system [20]. Furthermore, unlike mouth breathing, nasal respiration has been shown to entrain electrical oscillations in the amygdala and hippocampus, stabilizing emotional and cognitive networks [10]. We propose that this potent physiological signal activates the thalamic centromedian (CM) nucleus [8], shifting the brain’s behavioral bias from tic generation to respiratory maintenance. Consequently, the robust reward value of deep nasal respiration “overshadows” the maladaptive “micro-rewards” generated by tics, effectively breaking the chain of negative reinforcement [21]. Clinically, this suggests that the NARA method does not merely distract the patient but may actively remodel reward-processing circuits, leading to the sustained improvement observed over months.

### Mechanistic insights

1. Anatomical Airflow Optimization: Nasal breathing ensures the soft palate advances, reducing oropharyngeal resonance that may exacerbate vocal tics [15].
2. Autonomic Stabilization: Tics have been associated with sympathetic overactivity [22]. Slow, deep nasal breathing can induce parasympathetic dominance, mitigating tic triggers [23, 24].
3. Sensory Modulation: Many children with TS have heightened sensory reactivity or premonitory urges. The rhythm of controlled nasal respiration likely diminishes hyperarousal, yielding fewer tics over time [10].

### Transition from immediate suppression to long-term remodeling

Clinically, we observed a distinct trajectory of improvement. In the initial stages, as soon as the NARA maneuver ceases, patients typically revert to habitual mouth breathing, resulting in the immediate recurrence of tics. This “rebound” confirms that the suppression is dependent on the active maintenance of the physiological airway configuration. However, consistent home training gradually resolves lip incompetence [25] and establishes a habit of closed-mouth nasal breathing during the intervals between training sessions. As this physiological respiratory pattern becomes the patient’s baseline state (“default mode”), tic severity gradually declines, suggesting a long-term stabilization of the CSTC circuits driven by the sustained “primary reward” of nasal respiration.

### Clinical implications

From a practical standpoint, the NARA method requires no specialized equipment and imposes minimal burden on families [11]. Children can perform it discreetly during school or social activities, potentially preventing tic escalation. Notably, 3 of our patients overcame school refusal—a testament to how better tic management can restore participation in academic and community life.

In pediatric populations, adherence and benefits of patient-centered and self-managed therapies such as the NARA method significantly depend on active parental involvement. Future research should systematically examine how parental support influences both the immediate and long-term outcomes of this novel therapeutic approach.

### Limitations and future directions

Despite the promising outcomes, this study has several limitations inherent to its retrospective case series design and small sample size (*N* = 8), which limit the generalizability of the findings. The absence of a control group prevents us from definitively quantifying the extent of placebo effects or the natural waning of tics.

Although conducting strict randomized controlled trials for behavioral interventions in severe pediatric Tourette syndrome involves significant practical and ethical challenges, we acknowledge that our uncontrolled design cannot confirm definitive causality or efficacy. Consequently, at this stage, our findings remain preliminary and hypothesis-generating. However, the consistent clinical observations presented here offer highly valuable insights that strongly justify further prospective investigations. Furthermore, it should be emphasized that the observed improvements cannot be attributed to pharmacological effects. The majority of our cohort (6 of 8 patients) remained free of tic-suppressing medications throughout the entire follow-up period. In the few cases involving pharmacological intervention, significant tic reduction had already been achieved via the NARA method prior to any medication changes, confirming that the respiratory intervention was the primary driver of symptom relief.

Furthermore, clinical observations suggest mechanisms distinct from simple placebo or attentional distraction. Critics might argue that the NARA method functions merely as a “distraction” task. Yet, unlike passive relaxation or gaming tasks that may divert attention, maintaining tight lip closure during NARA is a physiologically demanding neuromuscular task for these patients, who often exhibit baseline orbicularis oris weakness [11]. Furthermore, the suppression of tics operated on a strict “On/Off” basis: tics re-emerged almost instantaneously when the lip seal was broken or exhalation shortened. This immediate, mechanically-dependent reversibility suggests a biomechanical and physiological antagonism rather than a generalized psychological placebo effect.

Future prospective or randomized clinical trials (RCTs), ideally multi-institutional, are warranted to solidify the method’s efficacy and explore potential synergies with other interventions, such as Comprehensive Behavioral Intervention for Tics (CBIT). As noted in recent trends, wearable sensors or smartphone apps could assist in real-time breathing guidance and objective tic monitoring, opening avenues for larger-scale implementation and home-based training [26]. To further facilitate the correct acquisition of the technique and support daily home practice—especially given the constraints of limited outpatient consultation time—we have recently released instructional videos on public platforms (e.g., YouTube: https://www.youtube.com/watch?v=G8VumF87rWw). These videos visually demonstrate the “3s–2s–6s” rhythm specifically recommended for children. Although the audio is in Japanese, the visual modeling is universal, and auto-generated subtitles allow for international accessibility. Furthermore, adherence to the daily home practice was evaluated via parent reports without objective monitoring devices. It should also be noted that the instructional video (Supplementary Video 2) was developed post hoc to standardize future instruction and was not utilized during the study period of this cohort. Additionally, advanced imaging or neurophysiological studies are needed to clarify the precise neural pathways by which this specific nasal respiration technique stabilizes tic symptoms.

## Conclusion

The Nasal Airway Respiration Anti-tic (NARA) method—a simple regimen of lip-closed nasal breathing—was associated with both immediate and sustained tic reduction in 8 elementary school children with Tourette syndrome. By directly targeting habitual mouth breathing, NARA helps shift respiratory and autonomic patterns that physiologically antagonize tic generation. Clinically, the method’s success relied on active parental support, highlighting the value of a family-centered approach. Our data indicate potential psychosocial benefits, including facilitating a return to regular school attendance. Further large-scale, controlled research is needed to confirm these preliminary findings and advance the understanding of closed-mouth nasal deep breathing as a viable component of TS therapy.

## Supplementary Information

Supplementary Material 1. Video 1: Representative patients showing the NARA effect. Short clips illustrate each patient’s habitual breathing and their response during the NARA method at the initial visit, followed by their normal breathing at the final follow-up. A substantial reduction in both motor and vocal tics is clearly observed soon after initiating lip-closed nasal deep breathing, and long-lasting improvements remain evident.

Supplementary Material 2. Video 2: Instructional video for the NARA method. This video demonstrates the specific breathing rhythm recommended for children (3s inhalation, 2s breath-holding, and 6s exhalation). Visual cues (animations) are used to guide the timing. Note: The audio instructions are in Japanese, but the visual modeling is designed to be universally understood. This video is also available online at: https://www.youtube.com/watch?v=G8VumF87rWw.

## Data Availability

The raw data was provided solely for peer-review purposes and is not publicly available to protect patient privacy. However, anonymized patient videos (with mosaic processing) are provided as supplementary materials, for which written consent for publication was obtained from all legal guardians.
